# Investigation of an outbreak of acute respiratory disease in an indigenous village in Brazil: Contribution of Influenza A(H1N1)pdm09 and human respiratory syncytial viruses

**DOI:** 10.1371/journal.pone.0218925

**Published:** 2019-07-08

**Authors:** Andrey Moreira Cardoso, Paola Cristina Resende, Enny S. Paixao, Felipe G. Tavares, Yasmin N. Farias, Carla Tatiana G. Barreto, Lídia N. Pantoja, Fernanda L. Ferreira, André Luiz Martins, Ângela Barbosa Lima, Daniella A. Fernandes, Patrícia Machado Sanches, Walquiria A. F. Almeida, Laura C. Rodrigues, Marilda M. Siqueira

**Affiliations:** 1 Fundação Oswaldo Cruz, Rio de Janeiro, RJ, Brazil; 2 London School of Hygiene and Tropical Medicine, London, United Kingdom; 3 Instituto Oswaldo Cruz—Fiocruz, Rio de Janeiro, RJ, Brazil; 4 Universidade Federal Fluminense, Niterói, RJ, Brazil; 5 Universidade do Estado do Rio de Janeiro, Rio de Janeiro, RJ, Brazil; 6 Secretaria Especial de Saúde Indígena, Curitiba, PR, Brazil; 7 Secretaria Municipal de Saúde de Paraty, Paraty, RJ, Brazil; 8 Secretaria de Vigilância em Saúde, Brasília, DF, Brazil; The University of Hong Kong, CHINA

## Abstract

Analyses of the 2009 H1N1 influenza pandemic and post-pandemic years showed high attack rates and severity among indigenous populations. This study presents the characteristics of the first documented influenza outbreak in indigenous peoples in Brazil, that occurred from 30th March to 14th April 2016 in a Guarani village in Southeast Region. Acute respiratory infections were prospectively investigated. The majority of the 73 cases were influenza-like illness (ILI) (63.0%) or severe acute respiratory infection (SARI) (20.5%). The ILI+SARI attack rate (35.9%) decreased with increasing age. There was a high influenza vaccination rate (86.3%), but no statistically significant difference in vaccination rates between severe and non-severe cases was seen (p = 0.334). Molecular analyses of 19.2% of the cases showed 100% positivity for influenza A(H1N1)pdm09 and/or hRSV. Influenza A(H1N1)pdm09 was included in the 6B.1 genetic group, a distinct cluster with 13 amino acid substitutions of A/California/07/2009-like. The hRSV were clustered in the BA-like genetic group. The early arrival of the influenza season overlapping usual hRSV season, the circulation of a drifted influenza virus not covered by vaccine and the high prevalence of risk factors for infection and severity in the village jointly can explain the high attack rate of ARI, even with a high rate of influenza vaccination. The results reinforce the importance of surveillance of respiratory viruses, timely vaccination and controlling risk factors for infection and severity of in the indigenous populations in order to preventing disease and related deaths, particularly in children.

## Introduction

Literature on investigation into outbreaks of acute respiratory disease among indigenous populations is scarce worldwide [[1–9]]. Analyses performed during the 2009 H1N1 influenza pandemic and post-pandemic years showed high attack rates among indigenous communities in the Americas and the Pacific. Rates of severity, hospitalization and death were also high, varying from three to six times greater than those observed in their non-indigenous counterparts [6]. Greater severity of respiratory disease in indigenous people has also been observed in seasonal influenza [10].

In Brazil, outbreaks are reported through the official information system of the Ministry of Health (Sinan–NET) and it has been observed that data for diseases like influenza or other respiratory viruses are analyzed, but rarely published. As far as we know, there is only one publication describing the investigation of an acute respiratory outbreak among indigenous peoples [11]. This study was carried out in 2010, in the North Region, and showed high attack rates of influenza-like syndrome when compared to other native people worldwide [2, 6, 7]. Attack rates were greater in children under five years of age, who also presented a higher degree of severity, and higher rates of hospitalization and mortality. Laboratory investigation has confirmed the circulation of human respiratory syncytial virus (hRSV), parainfluenza 3, human bocavirus and human metapneumovirus [11]. The incidence of severe acute respiratory illness (SARI) in indigenous people was 4.5 times greater than that reported for the overall population during the 2009 influenza pandemic in Brazil [6].

This study presents the clinical-epidemiological and molecular characteristics of an acute respiratory infection outbreak which occurred in March-April 2016 in a Guarani indigenous village in Southeast Brazil.

## Materials and methods

This study was conducted within the scope of the National Influenza Surveillance Program of the Ministry of Health, as part of a global and federal public health policy for influenza control and prevention in Brazil. The cohort study was approved by the Brazilian National Ethics Council (Comissão Nacional de Ética em Pesquisa–CONEP n. 719/2010), the Research Ethics Committee at the Escola Nacional de Saúde Pública, Fundação Oswaldo Cruz (CEP/ENSP n. 160/10) and the National Indian Foundation (FUNAI). In line with Brazilian legislation requiring that research with Indigenous populations respects culturally distinct community protocols for authorizing research, the study was presented at an open meeting in the village and, after approval, leaders signed a Collective Informed Consent Form on behalf of the community. The authorization for individual participants was verbal and, in the case of minors, was given by their parents or guardians. Individual participants or their guardians were made aware that they could decline or withdraw from participation at any time.

### Study design

Cases of acute respiratory infection (ARI), reported during an outbreak in an indigenous Guarani village in Southeast Brazil, were prospectively investigated to estimate attack rates and severity by age group and characterize the epidemiological curve. Furthermore, biological samples were primarily collected for severe cases, as recommended by official influenza surveillance system of the Ministry of Brazil [12], and additionally, when possible, for other non-severe cases of ARI or influenza-like illness, to investigate genetic characteristics of the viral strains detected.

### Surveillance and outbreak setting

The study village was part of a birth cohort study, the main objective of which was to measure the incidence of ARI during the first year of life. A trained health team visited the households weekly to allow early identification of ARI cases. In the last epidemiological week (EW) of March 2016 (EW 13/2016), a rapid increase in the number and severity of ARI cases was observed beyond the target birth cohort study population. The fieldworkers were supported by the technical section of the Indigenous Health Subsystem, the municipal epidemiological surveillance service and by the research group in order to actively search for cases in all households, collect information on disease and laboratory samples, and help to control the outbreak.

The outbreak period was from 30th March to 14th April 2016 (EW 13/2016 to 16/2016) and occurred in a Guarani village located in the rural area of the municipality of Paraty, Rio de Janeiro, Southeast Brazil (23^o^15´20´´S, 44^o^ 39´26´´W). This indigenous village is bordered by a local road, on a bus route towards the beach, which makes daily contact between the village community and the non-indigenous population possible. The population of the village comprises 170 individuals, 47 families allocated in 43 houses. We defined ‘indigenous’ as individuals who lived in the village and were considered indigenous by the community. This population has a younger demographic composition than the general population in Brazil. A multidisciplinary health team (MHT), under the auspices of the National Indigenous Health Subsystem, provides daily primary health care in the village. More complex health care, including epidemiological investigations, are provided, complemented and supported by the Unified Health System at the municipal level and elsewhere.

### Case definition

Cases were defined as: (a) general ARI—at least one of the following signs or symptoms: cough, respiratory distress, wheezing, runny nose, sore throat or earache; (b) influenza-like illness (ILI)—fever measured or reported plus cough and/or sore throat, with onset within the last 7 days; (c) severe acute respiratory infection (SARI)—ILI case plus respiratory distress with or without hospitalization.

According to WHO and Brazilian Ministry of Health [13, 14], SARI is usually defined as an ILI case plus respiratory distress requiring hospitalization. In this study we included in the case definition of SARI a subset of ILI plus respiratory distress without hospitalization, because the access to hospital facilities in indigenous villages in Brazil can be influenced by cultural or organizational contexts. However, where relevant, the results were shown for global SARI and separately for the subsets of SARI with or without hospitalization, enabling the comparison with other studies.

### Molecular detection of respiratory viruses

Fourteen nasopharyngeal swab samples were collected and sent to the National Influenza Center, FIOCRUZ, Rio de Janeiro for the detection of etiological agent. Refrigerated samples were received and submitted initially to immunofluorescence assay [15]. For the molecular detection, viral RNA was extracted directly from clinical samples, according to the QIAamp Viral RNA Mini Kit (Qiagen) manual. Real time RT-PCRs were conducted using Centers for Disease Control and Prevention (CDC) protocols for influenza A and B and hRSV detection [15]. The genetic sequencing steps were performed for influenza and hRSV positive samples with a cycle threshold (ct) lower than 32 in the real time RT-PCR protocols [15].

The haemagglutinin (HA) and/or neuraminidase (NA) genes of the influenza A(H1N1)pdm09 positive strains detected were the target genes to characterize the genetic group and determine the viral match with the A/California/7/2009-like (H1N1)pdm09 strain (EPI_ISL_203615), which comprised the trivalent influenza vaccine (TIV) from 2010 to 2016 in the southern hemisphere [16]. The reverse transcription (RT) and the PCR for the HA and NA genes were performed according to protocols described previously [15, 17].

The hRSVs were typed as belonging to group A or group B using a multiplex RT-PCR assay. RT-PCR was performed with the OneStep RT-PCR kit (QIAGEN) according to a protocol described previously [18].

For both viruses Sanger sequencing protocols were performed with a BigDye Terminator v3.1 Cycle Sequencing Kit (Thermofisher Scientific) using specific primers for each gene fragment. Gene sequences were determined according to the reading from the Applied Biosystems 3130xl Genetic Analyzers (Thermofisher Scientific).

Phylogenetic analyses were conducted for the HA and NA genes of influenza A(H1N1)pdm09 and for the G gene of hRSV using the maximum-likelihood (ML) method to using the HKY+gamma, the best substitution nucleotide model estimated by JModelTest. Trees were observed and edited on FigTree (http://tree.bio.ed.ac.uk/software/figtree/). Influenza A(H1N1)pdm09 sequences obtained in this study were deposited in the Global Initiative on Sharing All Influenza Data (GISAID) database under the accession numbers EPI_ISL_271538, EPI_ISL_271539, EPI_ISL_274050, EPI_ISL_274051 and EPI_ISL_274061 and the hRSV strains in Genbank under the accession numbers (MH719234 and MH719235).

### Statistical analysis

Descriptive epidemiologic analyses were performed using STATA 15 and a histogram of disease onset was built. We calculated frequency and proportions of ARI cases by demographic, clinical and laboratory features, and treatment characteristics were presented. Median and interquartile range (IQR) values were calculated for age. Comparisons of proportion of cases were made by sex and age groups, using Pearson’s chi-square test. Comparisons of means were made using t-test. Similar analyses were made to compare proportion sex and mean age between vaccinated and non-vaccinated subjects. The overall and age-specific attack rates for ARI (all cases), ILI + SARI and SARI, as well as for mutually exclusive categories of ILI, SARI hospitalized and SARI non-hospitalized, by age and sex were calculated using denominator data from the local census, checked a few weeks after the outbreak, and were compared using Pearson’s chi-square test.

## Results

**Fig 1:**
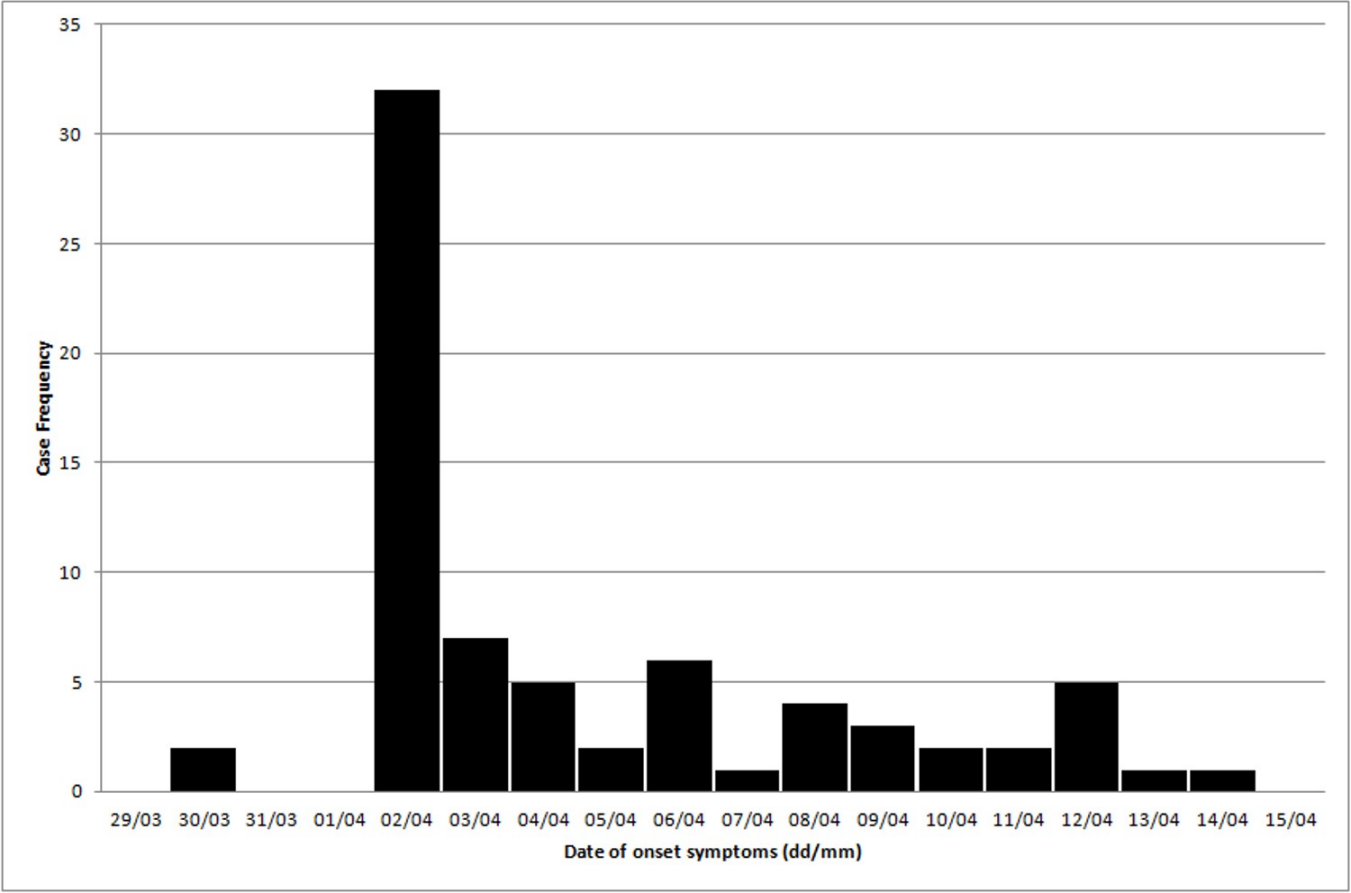
Epidemiological curve of the acute respiratory infection outbreak in the Guarani Indigenous village of Paraty Mirim, from epidemiological weeks 13 to 16. Rio de Janeiro, Brazil.

In the first six months of 2016, 97 cases of ARI were reported in 170 indigenous individual residents in the village, 73 (75.3%) of which were concentrated in the outbreak period, between EW 13 and 16 (Fig 1). The epidemiologic curve shows a sudden increase in ARI cases, with the last outbreak case reported on 14th April, 2016. In this study, we will further describe only the 73 cases that occurred during the outbreak period.

Cough and runny nose were the most frequent symptoms, occurring in 72/73 (98.6%) of the cases. Fever and respiratory distress were registered in 61/73 (83.6%) and 15/73 (20.6%) of the cases, respectively (Table 1). Other signs and symptoms described were shivering, diarrhea and oxygen blood saturation under 95% in oximetry.

**Table 1:** Demographic and clinical characteristics of acute respiratory infection cases during the outbreak in the Indigenous Guarani village of Paraty Mirim, Rio de Janeiro–Brazil, 2016.

Case characteristics		no.(%) / Median (IQR)*
Sex		
	Male	38 (52.0)
	Female	35 (48.0)
Age		
	Median (IQR)*	7.9 (3.2–17.0)
Influenza vaccination (yes + under vaccination age)		61 (89.7)
	Yes	58 (85.3)
	Under vaccination age	3 (4.4)
Signs and symptoms		
	Cough	72 (98.6)
	Fever	61 (83.6)
	Runny nose	72 (98.6)
	Respiratory distress	15 (20.6)
Case classification*		
	ARI non-ILI&SARI	11 (15.1)
	ILI	46 (63.0)
	SARI* (hospitalized or non-hospitalized)	15 (20.5)
	SARI (signs and symptoms, non-hospitalized)	8 (10.9)
	SARI (hospitalized)	7 (9.6)
	Ignored	1 (1.4)
Nasopharyngeal swab collection		
	Yes	14 (19.2)
Treatment		
	Antibiotics	59 (80.8)
	Antiviral (oseltamivir)	12 (16.4)
Hospitalization		
	yes	7 (9.6)

* IQR- Interquartile range, ARI non-ILI&SARI–acute respiratory illness non-influenza-like illness and non-severe acute respiratory infection, ILI–influenza-like illness, SARI—severe acute respiratory infection (with or without hospitalization), SARI (signs and symptoms, non-hospitalized)–subset of severe acute respiratory infection, non-hospitalized, SARI (hospitalized)—subset of severe acute respiratory infection, hospitalized.

The great majority of the outbreak cases were classified as ILI (46/73–63.0%), followed by SARI (15/73–20.5%). The attack rate of ARI during the outbreak period was 73/170 (42.9%), while the ILI+SARI, and separately the ILI and the SARI attack rates were 61/170 (35.9%), 46/170 (27.1%) and 15/170 (8.8%), respectively (Table 2).

**Table 2:** Acute respiratory infection age specific attack rates during the outbreak in the Indigenous Guarani village of Paraty Mirim, Rio de Janeiro–Brazil, 2016.

Age	N*	ARI* (all cases) n (%)	ILI+SARI n (%)	SARI* n (%)	Cases mutually exclusive*		
					ILI n (%)	SARI (hospitalized) n (%)	SARI (non-hospitalized) n (%)
All ages	170	73 (42.9)	61 (35.9)	15 (8.8)	46 (27.1)	7 (4.1)	8 (4.7)
<1	6	6 (100.0)	5 (83.3)	4 (66.7)	1 (16.7)	4 (66.7)	-
1–4	24	18 (75.0)	17 (70.8)	9 (37.5)	8 (33.3)	3 (12.5)	6 (25.0)
5–11	38	21 (55.3)	18 (47.4)	2 (5.3)	16 (42.1)	-	2 (5.3)
12–19	40	12 (30.0)	10 (25.0)	-	10 (25.0)	-	-
20–59	51	14 (27.5)	10 (19.6)	-	10 (19.6)	-	-
60 +	11	2 (18.2)	1 (9.1)	-	1 (9.1)	-	-

*N -population by age strata, ARI (all cases)–includes all cases of ARI occurred during the outbreak period, since all cases of ILI or SARI fulfil the ARI definition, ILI+SARI–includes severe (SARI) and non-severe (ILI) cases of Influenza-like Illness, SARI—includes only severe cases (with or without hospitalization), Cases mutually exclusive—shows the attack rates for the mutually exclusive categories of ILI, SARI hospitalized and SARI non-hospitalized.

There was no difference in the attack rates by sex for any of outcomes. However, the mean age among symptomatic people was lower than in non-cases (13.6 and 26.9 years, respectively p<0.0001). Almost one third of the cases (24/73 or 32.9%) occurred in children under 5 years old, while this age group represents only 17.6% of the village population. The age specific attack rates of ARI decrease with increasing age and this effect is stronger for ILI + SARI and SARI cases. Only two of the 15 severe cases occurred in individuals over 5 years old. Moreover, the attack rate of severe cases in under one-year olds was 1.78 times greater than that observed in the 1–4 years age group. Of those with signs and symptoms of SARI, 7/15 (46.7%) were hospitalized, all of these were children under 3 years old, and one infant progressed to death. The hospitalization rate was 7/73 (9,6%) and lethality rate was 1/73 (1.4%).

Of the total population, 9/170 (5.3%) were vaccination condition unknown. Of the 161 indigenous with known vaccination status, 136 (84.5%) had received the seasonal influenza vaccine in the last campaign (May 6, 2015) or in the vaccination routine during the period 2015/2016 (infants), before the outbreak, and three children (1.8%) were too young for vaccination. Thus, 86.3% (139/161) of those with known vaccination status would be supposedly protected against influenza. There was no difference in age (19.7 years vs. 24.5 years, respectively; p = 0.304) or sex (p = 0.762) between vaccinated and non-vaccinated subjects. There was no difference in vaccination rates between severe (SARI: 15/15, 100%) and non-severe (ARI or ILI: 46/53, 86.8%) cases (Fisher's exact p = 0.334). Furthermore, all 9 cases laboratory confirmed of influenza A(H1N1)pdm09 and eligible for vaccination at the time of the outbreak had received the vaccine in 2015.

Nasopharyngeal swab samples were collected in 14/73 (19.2%) of the cases and the molecular analyses showed 100% positivity: 5 (35.7%) for influenza A(H1N1)pdm09; 4 (28.6%) for hRSV; and 5 (35.7%) for both (Table 3). Thus, 71.4% of the laboratory-tested cases were confirmed as influenza A(H1N1)pdm09, or 13.7% (10/73) of the total number of cases identified during the outbreak. The presence of hRSV was confirmed in 64.3% of the laboratory-tested cases, or 12.3% (9/73) of all cases during the outbreak. There was no difference in the age between influenza A(H1N1)pdm09 and hRSV confirmed cases (p = 0.3276). Most of the confirmed cases of both influenza A(H1N1)pdm09 (7/10) and hRSV (7/9) had the same date of onset symptoms (Apr 2, 2016). All the 5 viral co-detection cases were severe. Co-detection was also observed in the case which resulted in death.

**Table 3:** Demographic and clinical data of patients with pathogens detected in the nasopharyngeal clinical sample during the outbreak in the Indigenous Guarani village of Paraty Mirim, Rio de Janeiro–Brazil, 2016.

PN*	Age	Sex	Vaccination TIV* (Days)	Case classification	Collection date (Days for the onset symptoms)	Pathogens detected by real time RT-PCR	Treatment^†^	Clinical outcome
1	60.7	M	Yes (328)	ARI*	Ignored	hRSV*	1	R*
2	5.8	M	Yes (326)	ILI*	Apr 8, 2016 (6)	FLU*	2	R
3	2.4	M	Yes (330)	SARI-NH*	Apr 8, 2016 (2)	FLU/hRSV	3	R
4	2.3	M	Yes (326)	SARI-NH	Apr 8, 2016 (6)	FLU/hRSV	3	R
5	2.3	M	Yes (230)	SARI-NH	Apr 6, 2016 (4)	FLU	2	R
6	1.6	M	Yes (266)	SARI-NH	Apr 8, 2016 (6)	hRSV	2	R
7	1.3	F	Yes (294)	ILI	Apr 8, 2016 (6)	hRSV	2	R
8	1.3	F	Yes (266)	SARI	Ignored	FLU	3	HR*
9	1.2	F	Yes (266)	SARI	Apr 4, 2016 (2)	FLU/hRSV	3	HD*
10	1.1	F	Yes (195)	SARI-NH	Apr 8, 2016 (6)	FLU/hRSV	3	R
11	9m*	M	Yes (62)	ILI	April 8, 2016 (5)	FLU	3	R
12	9m*	F	Yes (65)	SARI	April 8, 2016 (6)	FLU	3	HR
13	4m*	F	NA*	SARI	April 8, 2016 (6)	hRSV	3	HR
14	1m*	F	NA*	SARI	Apr 6, 2016 (4)	FLU/hRSV	3	HR

*PN–Patient number, m–age in months, TIV–trivalent influenza vaccine, NA—Non-applied (under minimum age for vaccination), ARI—acute respiratory illness non-influenza-like illness and non-severe acute respiratory infection, ILI–influenza-like illness, SARI—severe acute respiratory infection, SARI-NH—signs and symptoms of SARI, non-hospitalized, FLU—Influenza A(H1N1)pdm09, hRSV–human Respiratory Syncytial Virus, R–recovered; HR–hospitalized and recovered; HD–hospitalized and death.

†1—symptomatic, 2—antibiotic + symptomatic, 3—antibiotic + symptomatic + oseltamivir.

**Fig 2:**
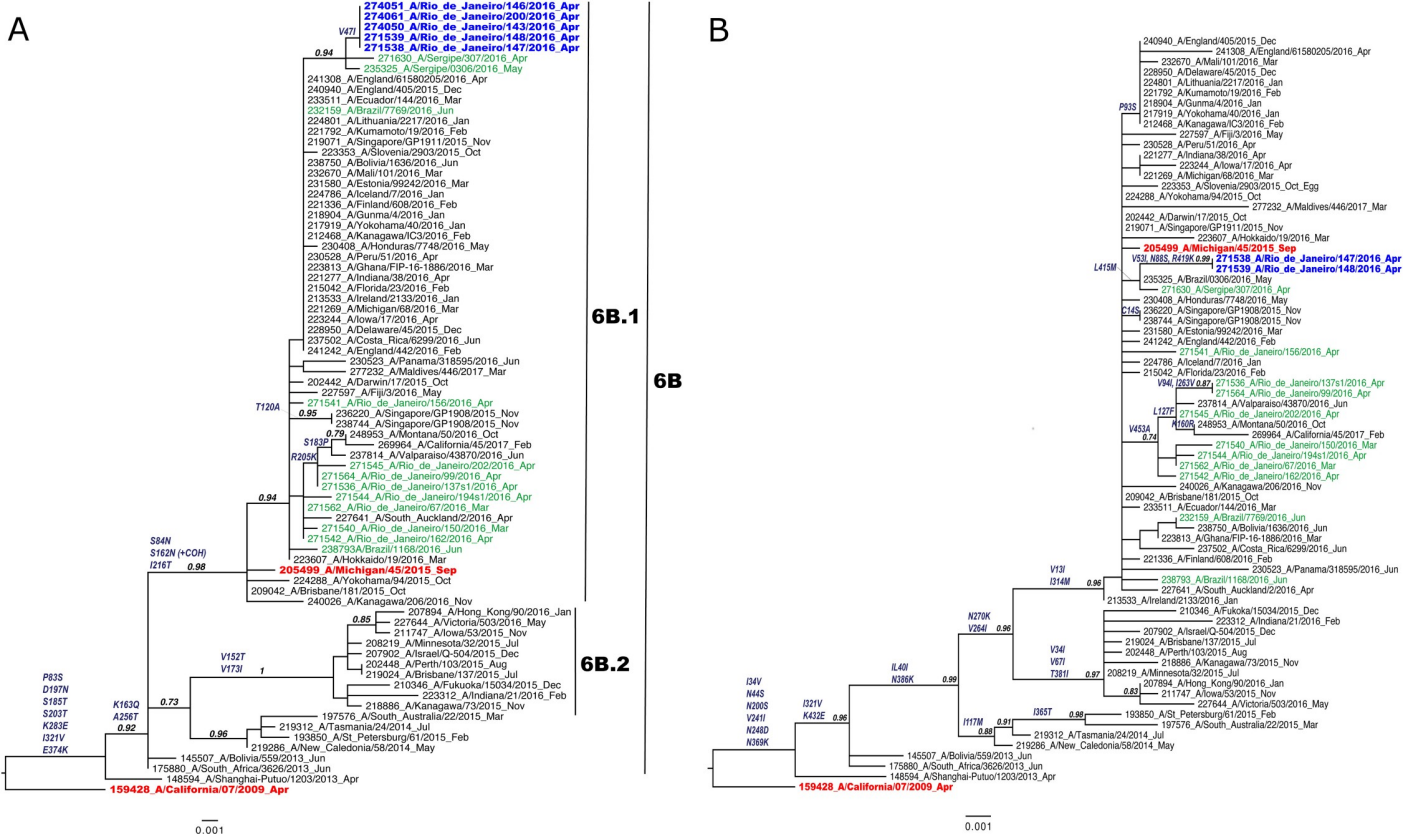
A–HA and B—NA. Maximum-Likelihood phylogenetic tree of hemagglutinin (HA) and neuraminidase (NA) genes of influenza A (H1N1)pdm09 strains Sequences obtained from the Guarani population are in blue, other Brazilian strains are in green, the vaccine strains are in red and other representative strains are in black. Amino acid substitutions in comparison with the vaccine strain A/California/07/2009-like chosen for the southern hemisphere from 2010 to 2016 are described in the branches.

**Fig 3:**
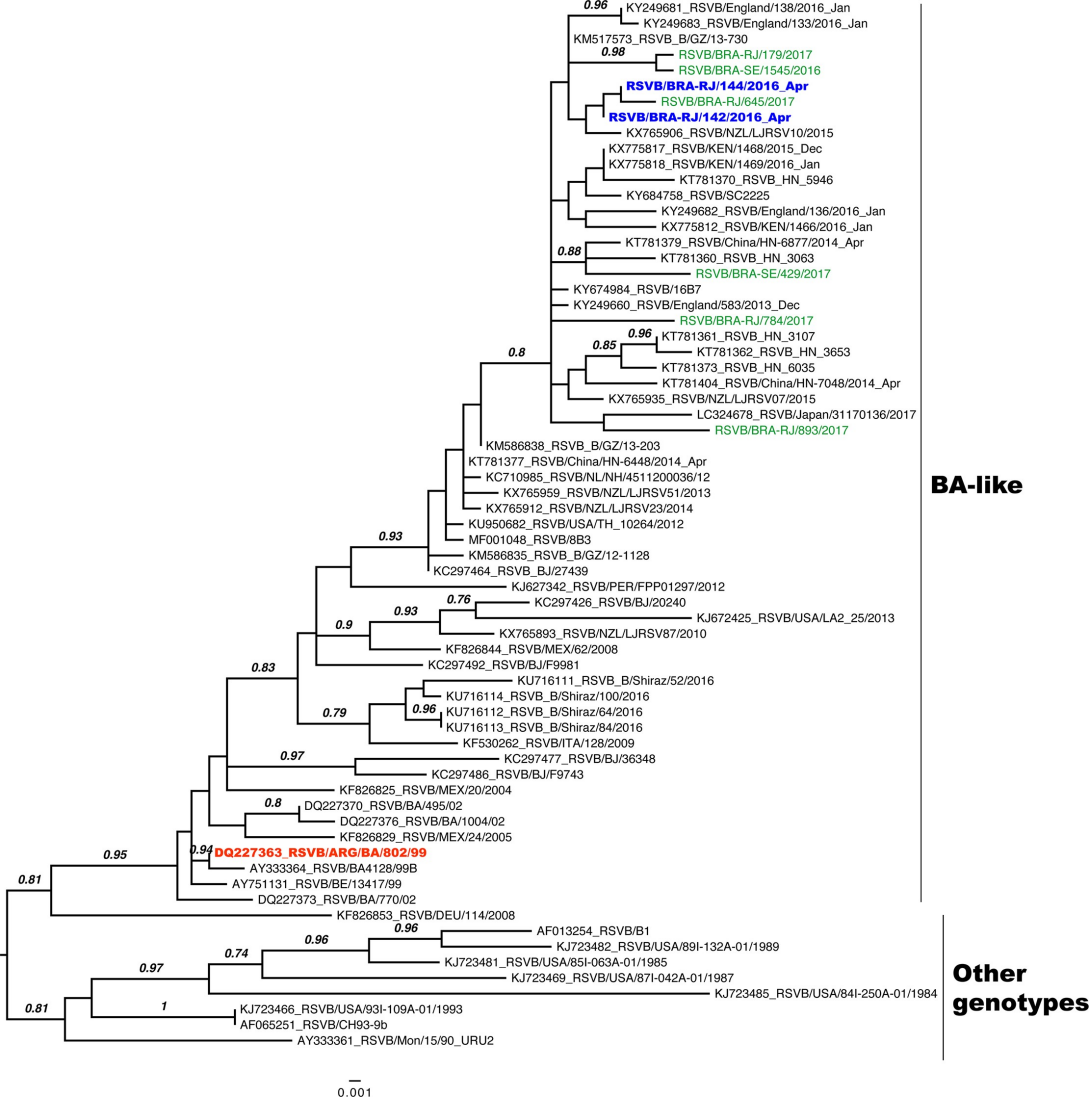
Maximum-likelihood phylogenetic tree of the G gene of respiratory syncytial viruses B Sequences obtained from the Guarani population are in blue, other Brazilian strains are in green, the reference strain with the insertion of 60 nucleotides characterized as BA genetic group is in red and other representative strains are in black.

The genetic group of the Guarani influenza A(H1N1)pdm09 strains was 6B.1, a distinct cluster of A/California/07/2009-like, with the 13 substitutions V47I, P83S, S84N, D97N, S162N, K163Q, S185T, S203T, I216T, A256T, K283E, I321V, E374K (Fig 2A-HA and Fig 2B-NA). We recovered two hRSV B strains by sequencing of the G gene which allowed us to establish the phylogenetic relationship between Guarani strains and other Brazilian and worldwide strains (Fig 3). They were clustered in the BA genetic group and some mutations (T107A, Y112H, R136T, T138S, P159del, K160del, I200T, L223P, T227N, S247P, T254I, T270I, V271A, I281T, S285F and H287Y) were observed when compared with the BA reference strain (DQ227363) of this genetic group (Table 4).

**Table 4:** Amino acid substitutions in the G gene of respiratory syncytial viruses B detected in Guarani population and in other Brazilian individuals in comparison with the reference strain DQ227363 with the insertion of 60 nucleotides (area highlighted in gray) characterized as BA genotype.

RSV B strains	Nucleotide substitutions in the G gene of RSV B																											
	76	99	107	112	118	136	138	142	159	160	200	214	216	223	227	247	252	254	267	270	271	272	281	285	287	295	303	312
DQ227363*	V	V	T	Y	I	R	T	Q	P	K	I	R	P	L	T	S	L	T	S	T	V	L	I	S	H	P	A	T
BRA-RJ/179/2017			A	H		T	S		del	del	T			P	N	P	F			I	A		T		Y		T	
BRA-SE/1545/2016		A	A	H		T	S		del	del	T			P	N	P	F	I	P	I	A		T		Y		T	
BRA-RJ/142/2016^†^			A	H		T	S		del	del	T			P	N	P		I		I	A		T	F	Y			
BRA-RJ/144/2016^†^			A	H		T	S		del	del	T			P	N	P		I		I	A		T	F	Y			
BRA-RJ/645/2017			A	H		T	S		del	del	T			P	N	P		I		I	A		T	L				
BRA-RJ/784/2017	I		D	H	T	T	S	K	del	del	T			P		P		I		I	A		T		Y			
BRA-SE/429/2017			A	H		T	S		del	del	T	G	L	P		P		L		I	A	F	T		Y	L		I

*DQ227363, accession number of the reference strain RSVB/ARG/BA/802/99 genotype A-like.

†RSV detected in Guarani population

Antibiotics were prescribed for 56/70 (80%) of cases and oseltamivir for 12/70 (17.1%) of the ARI cases. Oseltamivir was prescribed in 12/59 (20.3%) of the ILI or SARI cases (Table 2). It was also prescribed for 7/10 (70%) of the laboratory-confirmed influenza A cases. There was no record of palivizumab prescription.

## Discussion

This study described an outbreak of acute respiratory illness in an indigenous Guarani village in Southeast Brazil, with a very high attack rate of disease and co-detection of two respiratory pathogens—influenza A(H1N1)pdm09 and hRSV. The outbreak was of short duration, from 30th March to 14th April 2016, and occurred before the expected peak of seasonal influenza in the southeast region of Brazil, from May to August [19]. The attack rates were inversely related to age, as were the severity, hospitalization and lethality rates.

The epidemic curve of the Guarani village showed a propagated outbreak pattern, with a short duration, compatible with person-to-person infectious disease transmission in small populations. The concentration of almost 50% of the cases in a single day, suggests that the residents of the village were exposed to the disease for a short time and had similar incubation periods. In Guarani villages, close contact between individuals in community spaces for social, political or religious purposes is commonplace, making isolation unlikely, as reported in the other ARI outbreak among indigenous people in Pará [11]. During the outbreak investigation, no particular collective event that could explain the peak onset of symptoms on April 2 was identified. Possibly, the outbreak was limited firstly due to a reduction in the number of susceptible individuals and, secondly, due to treatment and general measures of disease control.

The seasonality of influenza in Brazil varies according to the geographical region and its climate characteristics; in the South and Southeast regions more intense activity is observed from May to August, following and overlapping the hRSV activity from March to June [20]. In 2016, the national influenza surveillance documented intense activity of influenza A(H1N1)pdm09 and hRSV, especially in southern Brazil, with the anticipation of the influenza epidemic season [19]. The same pattern was observed in Guarani cases confirming the concomitant circulation of both virus A(H1N1)pdm09 and hRSV and revealed the anticipation of influenza activity in the village.

The high attack rate (ARI: 42.9%; ILI+SARI: 35.9%) seen in the Guarani village resulted from the rapid and efficient spread of the disease, as reported in other small indigenous communities in French Polynesia [6] and Canada [7]. The ILI+SARI attack rate in the Guarani village was greater than the majority of that reported for other indigenous groups (13% to 20% in French Polynesia; 28% in Wallis and 38% in Futuna; 18% in New Caledonia [6]; 23% in an Aboriginal village in Australia [2]; 28.7% in a First Nations Community in Manitoba, Canada [7]) in the pandemic and post pandemic influenza 2009. It was also higher than the attack rate reported in Aboriginal and Torres Strait Islander in Australia (22.9%), estimated by immunity seroconversion after pandemic period [21]. But lower than the ARI attack rates reported in indigenous people in North Brazil (from 48% to 82%), in Pará, where no influenza virus was isolated, but hRSV, parainfluenza 3, human bocavirus and human metapneumovirus [11].

In general, indigenous populations with high attack rates of influenza A(H1N1)pdm09 are remotely located and naïve for influenza [2, 6, 7, 21]. Other indigenous populations with low vaccination influenza coverage still have shown high attack rates of disease in the post-pandemic period [2, 6, 7, 21]. Contrary to expectation, the Guarani group has permanent contact with the surrounding non-indigenous population, so is probably not naïve for influenza A(H1N1)pdm09 infection and other influenza strains. Additionally, the trivalent influenza vaccine (TIV), which has contained the influenza A(H1N1)pdm09 strain since 2010, is part of the Immunization Program for Native People in Brazil, and had 86.3% vaccination coverage in the 2015 seasonal campaign. These findings revealed the susceptibility of the village to a respiratory outbreak, even though they are not isolated and have high vaccination coverage, and suggests that the population might have attenuated immunity against seasonal influenza, possibly due to influenza antigenic drifts [22] and/or loss of vaccine-induced immunity over time.

The A/California/07/2009-like (H1N1)pdm09 strain was integrated in the influenza vaccine for the southern hemisphere from 2010 to 2016 [23]. Since the emergence of the influenza A(H1N1)pdm09 strain in 2009, some mutations in HA and NA were gradually incorporated by the virus population over the seasons. This culminated in the emergence and spread of the genetic group 6B.1, characterized by the HA substitution S84N, S162N with the addition of a glycosylation site, and I233T, in the northern hemisphere during the 2015–2016 epidemic season [24]. In Brazil, this genetic group, with important changes in antigenic sites in the HA protein, was introduced in 2016 [25] and may be responsible for the severe epidemic season of this year and for the outbreak in the Guarani population. After the severe 2016 epidemic season caused by 6B.1 H1N1 strains in the southern hemisphere and based on the genetic and antigenic analysis of the WHO influenza surveillance network, the H1N1 vaccine component was changed to the A/Michigan/45/2015-like (H1N1)pdm09 strain [22, 26]. In addition, the outbreak described here occurred before the influenza vaccination in 2016 and after almost one year after the last vaccine, resulting in a higher number of susceptible individuals.

Commonly detected in children, the hRSV also contributed to the ARI outbreak in the Guarani village. The phylogenetic analysis of these viruses revealed the presence of the hRSV-B genetic genotype BA-like, characterized by a 60-nucleotide duplication in the attachment (G) protein gene [27]. This genotype has been dominant worldwide and some genetic subgroups have been established in genotype BA after the first detection in 1999 in Buenos Aires, Argentina [28]. In this study specific mutations of hRSV-B strains were reported among the Guarani people which had not previously been reported, but these strains were closely identified with other Brazilian and worldwide hRSV- B strains from 2016 and 2017.

The severe cases were concentrated in children and none occurred in patients with risk factors such as chronic disease or pregnancy. It was also observed that all cases of viral co-detection were severe, although the clinical significance of this remains unclear [29, 30]. On the other hand, 5/10 (50%) of severe cases with viral confirmation were positive only for hRSV (2/10–20%) or influenza A(H1N1)pdm09 (3/10–30%), showing that the severity was not restricted to co-detection. The severity of the ARI outbreak observed in Guarani children has also been reported in other indigenous populations in Brazil [11] and elsewhere [2, 7]. The vulnerability of indigenous children in Brazil to ARIs has been discussed recently [31, 32]. Although the high attack rates of disease might be partly explained by the virus co-circulation, the severity of the disease in native people has been attributed to several conditions such as lack of tap water in households, overcrowding, undernutrition, exposure to tobacco and biomass smoke, limited access to healthcare, and increased genetic susceptibility [2, 6, 7, 33], most of which are reported to be highly prevalent among Guarani and other indigenous communities in Brazil [31, 34–36].

In Brazil, treatment with oseltamivir is formally recommended by the Ministry of Health in cases of ILI/SARI; however, this recommendation was only followed in 20% of the cases. Xiao and colleagues [34] verified the impact on the spread of the disease 24 hours after oseltamivir introduction in the Native population in Canada, preventing 962–1757 cases of disease and up to 114 medical evacuations. Better use of the available therapeutic arsenal and prioritizing early and widespread prescription of oseltamivir, could have been beneficial in reducing the severity and preventing the spread of disease.

We have provided a detailed description of the outbreak and its attack rates for acute respiratory syndromes by age, but we could not estimate precisely the specific attack rates for influenza A(H1N1)pdm09 and hRSV, since we have tested 19.2% of the cases. Based on laboratory confirmed cases, these would be about 30.6% and 27.6%, respectively, still high comparing to the attack rates described in the literature. The sample size for laboratory testing would be a plausible explanation for the lack of influenza vaccine effectiveness, if specific attack rate of influenza was biased. There was no statistically significant difference in age or sex between vaccinated and non-vaccinated subjects, and all laboratory investigated cases or severe cases were vaccinated against influenza, weakening this hypothesis. Then, we believe that our results suggest that the high attack rates can be strongly supported by the evidences of viral mutation, outbreak anticipation and high prevalence of risk factors.

The active surveillance of cases carried out in the village suggests that virtually all the incident cases were detected. Case definition based on reported signs and symptoms, although more sensitive for detecting cases, is more susceptible to overestimation, compared with other studies that used clinical signs measured, laboratory confirmation and/or antibody seroconversion. Nevertheless, our ILI and SARI definitions were more specific and presented similar results, reinforcing the precision of our findings.

## Conclusions and implications

The results have shown an early arrival of the influenza season before the 2016 influenza vaccination campaign. At the same time, it was confirmed the emergence and spread of a new influenza genetic group not covered by the 2015 vaccine [26] and an overlapping with the hRSV season. The confluence of those circumstances in an indigenous village where several risk factors for infection and severity are highly prevalent can explain the high attack rate of ARI even with a high rate of influenza vaccination, confirming the vulnerability of indigenous peoples to acute respiratory infections.

Thus, effective surveillance of respiratory viruses, timely vaccination and controlling risk factors for infection and severity of ARI in the indigenous populations are key public health measures for monitoring and preventing disease and related deaths, particularly in children. Establishing sentinel units in strategic villages with greater demographic mobility can help to identify influenza, hRSV and other virus circulation, and also genetic virus mutations. Influenza vaccination of pregnant women [37, 38], caregivers and household contacts of children too young to receive vaccine [39] seems to be a reasonable preventive strategy for younger children, since a severe laboratory-confirmed case of influenza A(H1N1)pdm09 was seen in children under the vaccination age.

Further studies on pathogen circulation in indigenous villages, and on maternal-infant passive protection, immune response to vaccination and vaccination effectiveness could bring new and robust evidence for indigenous health policy making and decisions regarding optimal timing for vaccination delivery.
